# Experimental Induction of Pulmonary Fibrosis in Horses with the Gammaherpesvirus Equine Herpesvirus 5

**DOI:** 10.1371/journal.pone.0077754

**Published:** 2013-10-11

**Authors:** Kurt J. Williams, N. Edward Robinson, Ailam Lim, Christina Brandenberger, Roger Maes, Ashley Behan, Steven R. Bolin

**Affiliations:** 1 Department of Pathobiology and Diagnostic Investigation, College of Veterinary Medicine, Michigan State University, East Lansing, Michigan, United States of America; 2 Large Animal Clinical Sciences, College of Veterinary Medicine, Michigan State University, East Lansing, Michigan, United States of America; 3 Diagnostic Center for Population and Animal Health, College of Veterinary Medicine, Michigan State University, East Lansing, Michigan, United States of America; University of Pittsburgh, United States of America

## Abstract

Gammaherpesviruses (γHV) are implicated in the pathogenesis of pulmonary fibrosis in humans and murine models of lung fibrosis, however there is little direct experimental evidence that such viruses induce lung fibrosis in the natural host. The equine γHV EHV 5 is associated with equine multinodular pulmonary fibrosis (EMPF), a progressive fibrosing lung disease in its natural host, the horse. Experimental reproduction of EMPF has not been attempted to date. We hypothesized that inoculation of EHV 5 isolated from cases of EMPF into the lungs of clinically normal horses would induce lung fibrosis similar to EMPF. Neutralizing antibody titers were measured in the horses before and after inoculation with EHV 5. PCR and virus isolation was used to detect EHV 5 in antemortem blood and BAL samples, and in tissues collected postmortem. Nodular pulmonary fibrosis and induction of myofibroblasts occurred in EHV 5 inoculated horses. Mean lung collagen in EHV 5 inoculated horses (80 µg/mg) was significantly increased compared to control horses (26 µg/mg) (*p* < 0.5), as was interstitial collagen (32.6% ± 1.2% vs 23% ± 1.4%) (mean ± SEM; p < 0.001). Virus was difficult to detect in infected horses throughout the experiment, although EHV 5 antigen was detected in the lung by immunohistochemistry. We conclude that the γHV EHV 5 can induce lung fibrosis in the horse, and hypothesize that induction of fibrosis occurs while the virus is latent within the lung. This is the first example of a γHV inducing lung fibrosis in the natural host.

## Introduction

Idiopathic pulmonary fibrosis (IPF) is a poorly understood respiratory disease of humans. In the United States of America the prevalence of IPF ranges from 14 to 27.9 cases per 100,000 individuals per year, making it is one of the more prevalent interstitial lung diseases[1]. Confounding these many cases is the lack of efficacy of most therapeutics for the disease; this lack of therapeutic options is associated with a 5-year mortality of between 50-70%[2]. The indolent nature of the disease, with its high mortality, unknown cause(s), and poorly understood pathogenesis, and the lack of animal models that recapitulate the progressive clinical course and pathology of IPF has hampered progress in management of the disease. 

 Viruses, in particular the γ-herspesviruses (γHV), may have a role in pulmonary fibrosis, as has been suggested in experimental murine models of fibrosis, as well as in humans with idiopathic pulmonary fibrosis (IPF)[3–5]. Epstein-Barr virus is the human γHV most commonly associated with the development of lung fibrosis[4,6,7], while in mice the normally non-pathogenic γ-HV MHV 68 induces pulmonary fibrosis in Th2-biased IFNγR^-^/^-^ mice[8,9]. The role the virus plays in the resultant increase in lung collagen in infected mice remains unclear. Recent data showing that latent pulmonary MHV 68 infection in wild-type mice enhances lung fibrosis in animals challenged with bleomycin or fluorescein isothiocyanate, suggests that an active lytic infection is not requisite for lung fibrosis to ensue[10,11].

 While experimental data in mice suggest that γ-HV may contribute to lung fibrosis, laboratory mice are often highly inbred, and are not the natural host for MHV 68[12,13], thus lung infections in such animals may not reflect the interplay between viruses and an outbred natural host as in humans with EBV infection. In particular, it remains unknown whether or not γ-HV are capable of initiating pulmonary fibrosis in their natural host on their own or if, as the murine data mostly suggests, they act as co-factors along with other external agents and host-specific factors in driving fibrosis in the lung. 

 Recently we identified and described equine multinodular pulmonary fibrosis (EMPF), a rare spontaneous progressive fibrosing lung disease in horses associated with lung infection with Equid Herpesvirus 5 (EHV 5), a γ-HV of horses[14,15]. The disease has a characteristic clinical presentation, typified by low-grade fever, weight loss, and progressive exercise intolerance, along with radiographic evidence of nodular pulmonary interstitial fibrosis[15]. EHV 5 is consistently detected within the lungs of horses with EMPF[14]. Additional evidence for γ-HV infection includes successful isolation of the virus from affected lung, and visualization of intranuclear herpesvirus-like inclusion bodies within alveolar macrophages through both light microscopy and electron microscopy[14]. 

 While interstitial pneumonia has been reported in cats and baboons in association with lytic pulmonary α-herpesvirus infection[16,17], the recognition of an association between lung infection with EHV 5 and EMPF is the first example of a spontaneous γ-HV-associated progressive fibrosing lung disease occurring in an outbred population of the natural host species, and provides an opportunity to better understand the role that infections with γ-HV may play in progressive lung fibrosis in humans.

 The purpose of the current study was to test the hypothesis that isolates of EHV 5 obtained from spontaneous cases of EMPF would induce pulmonary fibrosis after being inoculated into the lungs of horses. This study provides evidence that EHV 5 is the cause of EMPF and is the first to show that a pulmonary γ-HV infection in the natural host can lead to lung fibrosis in an outbred group of the natural host species without additional known coincidental lung insults. Investigations into the biology of EHV 5 lung infection may provide insights into the role that γ-HV infections play in human pulmonary fibrosis, especially with EBV where virus infection is common in the natural host, yet the disease remains relatively rare.

## Materials and Methods

### Ethics Statement

The Institutional Animal Care and Use Committee at Michigan State University approved of all experiments utilizing animals reported in this study. 

### Animals

Eight female mixed-breed horses were purchased for the study. Six horses were designated (E1 - E6) for inoculation with EHV 5 and 2 horses (C1, C2) were used as sham-inoculated control animals. The age of the horses, determined by examination of the dentition, ranged from 10-26 years (see Table 1). The animals were housed outdoors on pasture, with access to open-sided shelter during inclement weather. The horses inoculated with EHV 5 were housed on pasture isolated from the non-inoculated control horses, as well as from other horses. The horses were acclimated to their holding facilities for 30 days prior to the beginning of the experiment.

**Table 1:** Detection of EHV-5 in blood obtained on day 0 before infection and on sampling days 4 to 49 after infection.

Day PI	C1_10y_	C2^a^_16y_	E1^*^_19y_	E2_20y_	E3_17y_	E4^**^_17y_	E5_26y_	E6_16y_
0	−	V_C_	V^§^	V	−	−	−	−
4	−	V_C_	ND	−	−	B	−	−
7	−	V_C_	ND	−	−	−	−	−
11	−	V_C_	ND	V	−	−	−	−
14	−	V_C_	ND	−	−	−	−	−
21	ND	V_C_	ND	−	−	−	V	−
28	ND	V_C_	ND	−	B, V_C_	−	−	−
35	ND	V_C_	ND	−	−	−	−	−
42	ND	V_C_	ND	V	−	−	−	−
49	ND	V_C_	ND	−	−	−	−	−

^a^ Horse C2 was naturally infected with an EHV-5 that was designated as V_C_. This horse was in contact with other horses in the study before separate housing was initiated.

* Horses E1, E2, and E3 were experimentally infected with an EHV-5 designated as A

** Horses E4, E5, and E6 were experimentally infected with an EHV-5 designated as B -; negative; ND: not done; V: several genetically identifiable EHV-5 were detected in blood from the horses, those viruses are collectively designated as V. The age of all horses in years (y) is denoted in subscript.

### Pre-Inoculation Evaluation of Horses

To select horses for experimental inoculation, samples of serum and whole blood were obtained from 22 candidate horses. Sera were tested for antibody that bound viral antigens produced by EHV 5 as well as for antibody that bound antigens from the related equine γ-HV EHV 2. Detection of antibody was done using rabbit kidney (RK13 (ATCC® CCL37™)) cell cultures that had been biologically cloned free of contaminating bovine viral diarrhea virus prior to use[18]. At 4 or 5 days after infection of RK13 cell cultures with EHV 2 or EHV 5, respectively, the cultures were fixed with buffered acetone and serial 2-fold dilutions of the sera were tested for antibody using an immunoperoxidase staining procedure[18]. From this population 8 animals were selected based on having the lowest titers of antigen binding antibody. . 

To collect pre-exposure bronchoalveolar lavage (BAL) fluid from the 8 study horses, a 3m endoscope was passed into the lungs via the nares and wedged into a peripheral bronchus. Three sequential 100ml aliquots of sterile PBS were instilled into the lung and retrieved by aspiration. Recovered lavage fluid from each horse was pooled, and the volume was recorded. Total cell counts were performed on the lavage fluid using a hemocytometer, and differential cell counts were obtained by examining 300 cells on modified Wright’s stained cytospin preparations (data not shown). 

Approximately 50 ml of BAL fluid and 10 ml of whole blood from each horse were centrifuged at 1700 rcf for 10 minutes at 4° C to pellet cells in the BAL and to form a buffy coat layer within the whole blood. After centrifugation, the BAL fluid was decanted and the remaining cell pellets were suspended in 1 ml of Bovarnick’s buffer and frozen at -80° C until used in polymerase chain reaction (PCR) assays for detection of viral genome (DNA). 

Peripheral blood mononuclear cells (PBMC) were obtained from 10 ml of blood collected into a heparinized tube. The blood tube was centrifuged at 1,700 rcf for 10 minutes and the buffy coat layer was aspirated, washed once in isotonic PBS, and pelleted by centrifugation at 270 rcf for 12 min at 4° C. Contaminating red blood cells were removed from the PBMC by flash lysis using sterile cell culture grade water followed by addition of a 2x concentration of PBS. The remaining PBMC were pelleted by centrifugation, as above. The final cell pellet was suspended in 1 ml of Bovarnick’s buffer and frozen at -80° C until used in PCR assays. 

### Viral inocula

Two viral isolates of EHV 5, designated ‘A’ and ‘B’, were used in this study. Both isolates were obtained from lung tissue of horses diagnosed with EMPF. The two isolates were readily differentiated by nucleic acid sequence within the viral gB gene. Viral stock solutions were made from the fifth passage of each virus in RK13 cells. At 6 days after infection with virus, flasks of RK13 cells were frozen and thawed and 100 ml of cell culture supernatant for each virus was clarified by centrifugation at 1,850 rcf for 30 minutes. The clarified supernatants were decanted into new vessels and a 1 ml aliquot of each virus was removed and placed in a vial. The clarified supernatants and 1 ml vials were frozen at -80 C. After 48 hours, the 1 ml vials were thawed and serial 10-fold dilutions were inoculated in replicates of four onto semi-confluent monolayers of RK13 cells grown in 24-well cell culture plates. After 1 hour, cell monolayers were overlaid with fresh McCoy’s 5A medium supplemented to 6% with virus-free fetal bovine serum. The medium was changed at 3 day intervals. After 9 days the cells were passaged to new 24-well cell culture plates. After an additional 9 days, the concentration of virus was estimated by observing the cell monolayers for cytopathic effect. This observation was confirmed by testing positive and negative wells for viral DNA using a viral glycoprotein H gene (gH)-specific PCR assay as described below.

### Inoculation of horses

**Figure 1:**
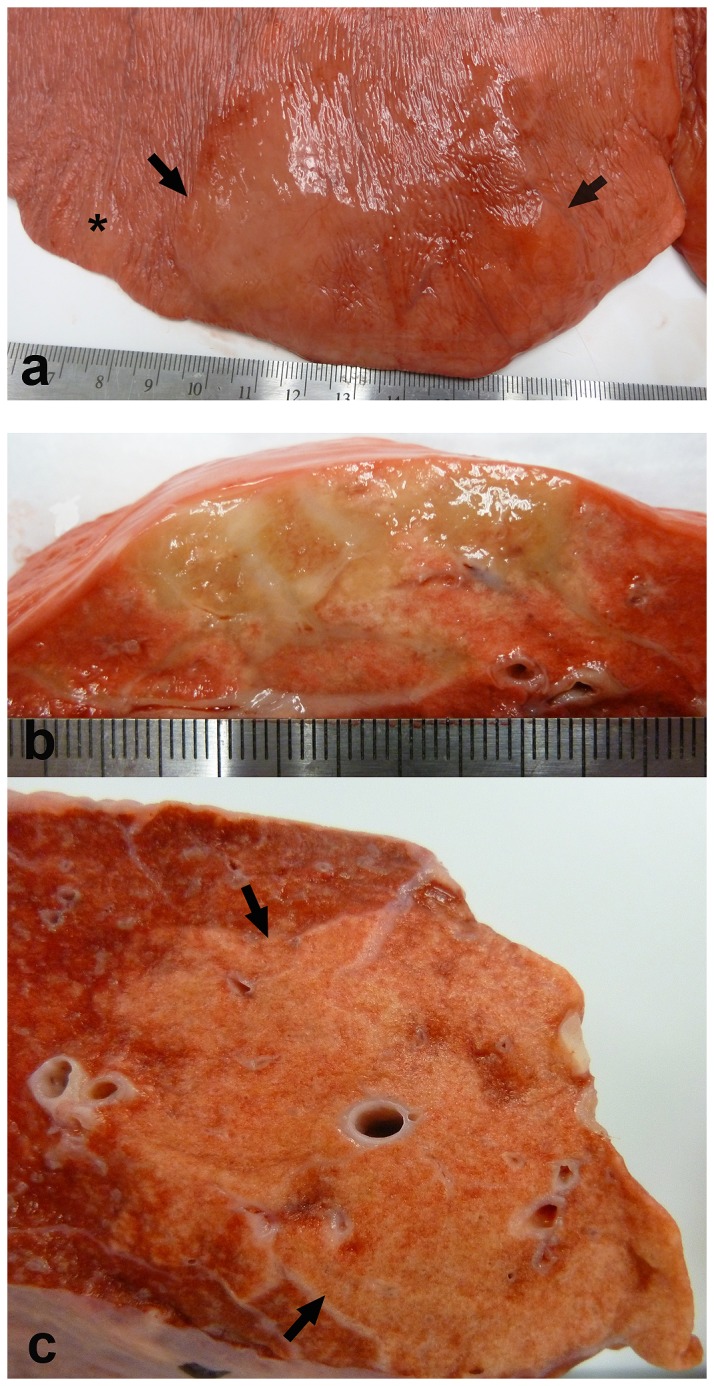
Experimental EHV 5 infection; gross pathology (horse E3, E4). Nodules of fibrosis (A, between arrows, and at asterisk; horse E4) were evident beneath the pleura (cut section, B; horse E4). The nodules of fibrosis extended into the underlying alveolar parenchyma (B,C; horse E3,E4 respectively).

To inoculate the horses, a 3m endoscope was passed via the nares into the lobar bronchus of the accessory lung lobe. The accessory lung lobe was chosen for inoculation because of its accessibility and its gravity-dependence within the thoracic cavity, making retention of the inoculum within the lobe likely. Three horses each received virus A or virus B in 20 ml of inoculum that was adjusted to contain a total of approximately10^6^ cell culture infectious viral particles, or 10^8^ PCR detectable units of viral DNA (Figure 1A and 1B). The inoculum was instilled via a polyethylene catheter passed through the biopsy port of the endoscope. The two control horses were sham inoculated with uninfected RK13 cell culture lysates.

### Monitoring the horses

All horses were qualitatively assessed as to their well being on a daily basis during the experiment, with particular attention paid to feed intake, body condition, and activity level. Each animal’s body temperature, pulse, and respiratory rate were recorded on the day of virus inoculation, and on days 1, 2, 3, 4, 5, 6, 7, 11, 14, 21, 28, 35, 42, and 49 post-inoculation. Blood was collected from the jugular vein on days -2, 0, 4, 7, 11, 14, 21, 28, 35, 42, and 49 for a complete blood count, and for measurement of serum fibrinogen and total protein levels. In addition, PBMC were harvested from the blood samples by the aforementioned methods, except that the PBMC were suspended in 1 ml of McCoy’s 5A medium supplemented to 6% with virus-free fetal bovine serum. Aliquots of PBMC were used for virus isolation and for isolation of DNA that was subjected to the viral gH gene-specific PCR assay. 

### Necropsy and Tissue Collection

The horses were euthanized using an overdose of pentobarbital administered into the jugular vein. Horse C1 was euthanized at day 14 PI; horse C2 at day 97 PI; horses E1 and E2 at day 98; horse E4 and E6 at 105 days PI; and horses E3 and E5 at 108 days PI. The horses underwent a complete postmortem examination, and tissues were collected for histopathology and virologic analysis by PCR and virus isolation. The following tissue sites were collected: bronchial lymph node, mesenteric lymph node, submandibular lymph node, spleen, bone marrow, respiratory nasal mucosa, kidney, liver and lung. Sections of tissue were collected from the accessory lung lobe and the left and right cranial and caudal lung fields. When gross lesions were observed, multiple samples were taken from lung lesions as well as the surrounding grossly normal lung. Stomach, small intestine, large intestine, and myocardium were collected for histopathology only. All tissues samples designated for histopathology were immersed in 10% neutral buffered formalin for a minimum of 24 hours prior to processing for histopathology. All tissue samples designated for virus isolation and PCR assay were frozen at -80 C until analyzed.

### Virus Isolation

Virus isolation was attempted on PBMC from all samples of blood obtained from the horses before and after inoculation with EHV 5 and on all tissues harvested post mortem. To process the tissues for viral isolation, 2 to 3 grams of thawed tissue was minced using a razor blade and transferred to a 15 ml conical tube containing 8 copper ball bearings (6 mm in diameter) and 7 ml of chilled Bovarnick’s buffer. The contents of the tube were agitated at full power for 1 minute, using a vortex. Approximately 1 ml of the resulting homogenate was removed, placed in a microcentrifuge tube, and centrifuged at 20,000 rcf for 10 minutes. The clarified supernatant was then passed through a 0.45 μm syringe filter onto a 75% confluent monolayer of RK13 cells in 25 cm^2^ culture flasks. After 30 minutes, 5ml of McCoy’s 5A culture medium, supplemented with 6% virus-free fetal bovine serum, was placed on the cells. The medium was changed every 3 days for 9 days, then the inoculated culture was split 1:2 and the resulting duplicate cultures were maintained for an additional 9 days. Approximately half of the cells in each of the duplicate cultures were passaged at 9-day intervals to form new duplicate cultures through 6 cell passages. At each change of culture medium, the cells were observed for cytopathic effect, characterized by formation of multinucleated giant cells. After the final cell passage, DNA was extracted from the cells using a commercially available kit (DNeasy Blood and Tissue Kit, Qiagen, Valencia, CA) and PCR assays targeting the viral glycoprotein H (gH) and glycoprotein B (gB) genes of EHV 5 and the gB gene of EHV 2 were performed as described below.

### PCR assays

Several PCR assays were used for detection of equine γ-HVs on PBMC and BAL obtained from horses before inoculation with EHV 5, on all samples of PBMC obtained after inoculation with EHV 5, and on all tissues harvested post mortem. DNA was extracted from thawed suspensions of PBMC or cells pelleted from BAL fluids using a rapid alkaline polyethylene glycol-based method that allowed direct PCR[19]. For detection of viral DNA in tissues obtained post mortem, and for derivation of viral nucleic acid sequences, DNA was extracted from 200 μl of the tissue homogenates, using the DNeasy® Blood & Tissue Kit (Qiagen Inc, Valencia, CA), as recommended by the manufacturer. 

The nucleic acid sequences of the PCR primers and the PCR reaction conditions are provided as File S1. The initial PCR assays used to screen horses for presence of virus before inoculation with EHV 5 targeted the viral glycoprotein B (gB) gene of EHV 2 or the viral glycoprotein H (gH) gene of EHV 5[20,21]. The assays were modified from their original descriptions to allow for reaction conditions that accommodated use of a different reagent mix (Promega GoTaq Green, Promega, Madison, WI). Additionally, the assay for gB gene of EHV 2 was changed from the original hemi-nested PCR to a PCR assay that employed only the original set of first round PCR primers. The PCR assay for the viral gH gene of EHV 5 was changed from a standard procedure to a touchdown procedure. A second PCR assay for EHV 5 that targeted the viral gB gene also was done on all samples from the horses. All of the aforementioned PCR assays (Table 1) were performed on DNA from all tissues harvested at post mortem examination of the horses (Figure 1C is an example). Nucleic acid sequences were derived for select regions of the viral gB gene, as described previously For select horses, PCR assays were performed that targeted the viral DNA packaging protein gene of equine γ-HVs or the generic herpesvirus DNA-directed DNA polymerase gene[22–24]. 

### Nucleic Acid Sequencing

Viral DNA detected in samples from horses was analyzed using nucleic acid sequencing of select regions of the viral gB gene of EHV 5. To accomplish this, several PCR primers were designed from publicly available nucleic acid sequences of the gB gene. Those PCR primers were used to derive the nucleic acid sequence of the gB gene for each of the viruses used as inocula in this study (virus A, GenBank accession # KC715730 and virus B, GenBank accession # KC715731) and for an EHV 5 that was repeatedly detected in one of the control horses ( virus Vc KC715732 ). Conserved regions within the gB gene were exploited to obtain PCR primers that amplified an approximately 850 base sequence that started at base 54 of the open reading frame of the gB gene. Additional PCR primers were used to derive the sequence for approximately 660 bases located just downstream of the above 850 base sequence. This PCR amplicon terminated between base 1554 and 1579 of the gB gene, depending on the EHV 5 virus. For derivation of nucleic acid sequence, a high fidelity Taq polymerase was used. PCR amplicons of the appropriate size were excised from agarose gels after electrophoresis, purified with QIAquick Gel Extraction Kit (Qiagen Inc, Valencia, CA), and eluted in 30ul of nuclease-free water. Aliquots of the DNA were then directly sequenced in both directions at the Research Technology Support Facility, Michigan State University. Chromatograms from each sequence submission were edited, assembled, aligned, and trimmed using commercial software programs (Sequencher™, Gene Codes Corporation, Ann Arbor, MI; and Clone Manager Professional Suite, Science and Education Software, Cary, NC). 

### Viral Neutralization

Viral neutralization (VN) assays were done on samples of sera obtained from the horses immediately before viral inoculation (Day 0) and on days 21, 42, and 49 PI.  Briefly, starting with a 1:8 dilution of serum in McCoy’s 5A medium, serial 2-fold dilutions of each serum were made in duplicate in a 96 well microtitration cell culture plate.  Approximately 35 cell culture infective doses (CCID_50_) of virus A or approximately 60 CCID_50_ of virus B (as calculated from back titers of virus using the Spearman-Kärber method) were added to each well.  The plates were incubated at 37 C for 1 hour, then the mixtures of virus and diluted sera were removed and inoculated onto 75% confluent monolayers of RK-13 cells in 96 well plates.  The culture medium over the RK-13 cells had been removed immediately before inoculation. After 24 hours, 100 μl of medium was added to each well to bring the total volume in each well to 260 μl. The cell culture medium was removed and replaced with fresh medium every 3 days for 9 days.  Absence of cytopathic effect at 12 days was used as evidence of viral neutralization. The reciprocal of the highest dilution of sera in the duplicate plates that neutralized virus was used as the viral neutralizing antibody titer.

### Histopathology

Tissues were routinely processed and embedded in paraffin, sectioned at 6 µm, and stained with hematoxylin-eosin. The lung collagen content was assessed morphometrically (see below) in serial sections from these same regions of the lung using picrosirius red, a collagen-specific histochemical stain[25,26].

### Immunohistochemistry

To assess for the presence of myofibroblasts, 6 µm sections of lung from spontaneous EMPF, experimental EHV 5 infected horses and control horses were deparaffinzed in xylene and rehydrated in a graded series of ethanol before being incubated overnight at 4° C with a 1:100 dilution of mouse monoclonal antibody against α-smooth muscle actin (Dako Corporation; Carpenteria, CA). This was followed by incubation with appropriate secondary antibody, avidin-biotin conjugated horseradish peroxidase as per manufacturer’s instructions (Vector Laboratories; Burlingame, CA) and diaminobenzidine (Sigma Chemical; St. Louis, MO). Tissue sections incubated with either PBS or mouse IgG in place of the primary antibody served as negative controls. 

To localize EHV 5 within the lungs of the horses, polyclonal rabbit anti-EHV 5 serum was generated by injecting rabbits with either of the two EHV 5 isolates used in the equine exposure. Serum was collected from the infected rabbits following seroconversion to the virus and incubated with sections of lung as outlined above at a concentration of 1:100. This was followed by incubation with appropriate secondary antibody conjugated with avidin-biotin horseradish peroxidase as per manufacturer’s instructions (Vector Laboratories; Burlingame, CA) and diaminobenzidine (Sigma Chemical; St. Louis, MO). Tissue sections incubated with either PBS or rabbit IgG in place of the primary antibody served as negative controls. To confirm the identity of the apparently infected honeycomb epithelial cells within the nodules of fibrosis immunohistochemistry was performed using an antibody against epithelial cytokeratin intermediate filaments (MNF 116, Dako Corporation; Capenteria, CA) at 1:100 followed by visualization as above and counterstaining with hematoxylin.

### Quantification of lung collagen

Quantitation of lung collagen was performed using two techniques: the Sircol assay (Biocolor, UK) and morphometry. To perform the Sircol assay duplicate sets of 100 mg of lung from each horse was used in the analysis; the tissue analyzed did not include large airways or blood vessels. The methodology followed that recommended by the manufacturer. Briefly, the tissues underwent overnight acid-pepsin digestion followed by collagen isolation and concentration before the samples were incubated with the collagen binding dye Sirius red. Collagen concentration was determined using a Bio Tek ELx 808 plate reader (Bio Tek, Winooski, VT) reading absorbance at 570nm; the data was expressed as µg collagen per mg wet tissue. 

To morphometrically quantify lung collagen we used serial histologic sections from the lung regions, picrosirius red staining, and histologic examination[25,26]. To quantify lung collagen the histology slides were scanned and digitalized at 20x magnification using the virtual slide system VS110 (Olympus, Hicksville, NY). Sub-sampling of images within the tissues as well as morphometric analysis was carried out using NewCast software (Visiopharm, Hoersholm, Denmark). Briefly, an automated systematic uniform random sampling of approximately 70 images was performed on each of the digitalized slides at a 20x magnification. A point grid with an area per point of 1690 mm^2^ was then super imposed over the images. Points hitting the parenchymal (alveolar) tissue were counted as well as the fraction of points hitting collagen positive areas. Points encompassing airspace, vasculature, interlobular septa and pleura were excluded from the evaluation; 300 to 400 points were counted on average per section. The percentage of collagen in the parenchyma of lung sections was then estimated by dividing the number of points hitting collagen areas by the number of total parenchymal tissue points. The difference of percent parenchymal collagen in lung regions of EHV 5 infected (n=12) and control horses (n=6) was analyzed using a t-test. Data are presented as a mean ± standard error of the mean (SEM).

### Statistical Analysis

An unpaired t-test was used to compare mean lung collagen concentration between control and EHV 5 inoculated horses as determined using the Sircol assay after log conversion of the data. A *p* value < 0.05 was considered significant. For the morphometric collagen determination the difference of percent parenchymal collagen in lung regions of EHV 5 infected (n=12) and control horses (n=6) was analyzed using a t-test. Data are presented as a mean ± standard error of the mean (SEM). A *p* value <0.05 was considered significant.

## Results

### Pre-inoculation evaluation of horses

EHV 5 or EHV 2 DNA was not detected within cells isolated from the BAL fluid obtained from any horse prior to experimental exposure with virus. One of the control horses, C2, and 2 of the experimentally inoculated horses, E1 and E2, had detectable EHV 5 DNA within PBMC before experimental infection with EHV 5. Horses E1 and E2 also had detectable EHV 2 in their PBMC before inoculation with EHV 5. The titers of antigen-binding antibody ranged from 64 to 512 for both EHV 2 and EHV 5 for the 8 horses selected for the study, as determined by immunoperoxidase monolayer assay. For individual horses, the titers of antibody against EHV 2 or EHV 5 were within a 2-fold dilution of each other (data not shown). 

### Post-inoculation clinical monitoring

None of the experimentally infected horses developed an elevated body temperature during the post-inoculation monitoring period, nor did they exhibit outward clinical signs of disease that could be associated with a viral infection. Horse C1 developed severe lameness shortly after the study began and failed to respond to treatment. For humane reasons this animal was euthanized on day 14 of the study. There were no significant changes in any of the blood values for 5 of the 6 of the infected horses. The 6^th^ horse (E1) was intractable, so blood was not collected post-infection to provide for the safety of animal handlers. In a single horse (E2), neutrophil numbers fluctuated over the course of the experiment. In this animal, there was a moderate elevation in neutrophils detected at 4, 7, and 11 days post-infection (7.61 - 9.01 x 10^3^/µl), which dropped to within the normal range (1.9-7.4 x 10^3^/µl ) on days 14 and 21 post-infection, then elevated again on day 28 PI (9.44x 10^3^/µl), was normal days 35 and 42, and elevated on day 49 PI (7.68 x 10^3^/µl).

### Viral isolation and detection of viral DNA in blood

Virus was not isolated from any blood sample obtained from control or experimentally inoculated horses during the study. After viral inoculation, EHV 2 DNA was detected by PCR in PBMC from horse E2 on 6 occasions between days 4 and 49 (data not shown) and EHV 5 DNA was detected in 5 samples of blood that had been obtained from 4 horses between days 4 and 42 of the study (Table 1). However, nucleic acid sequence analysis indicated that the two strains of EHV 5 used for exposure were only detected twice and strains of EHV 5 other than those used for inoculation were detected on 3 occasions. 

### Viral Neutralization

All but one horse had neutralizing antibody titers that ranged from 8 to ≥1,024 against EHV 5 isolates A or B prior to inoculation (Table 2). One horse lacked detectable concentrations of neutralizing antibody against EHV 5 isolate B prior to inoculation. In general the experimentally inoculated horses showed a modest rise in neutralizing antibody titer through day 49 post-inoculation, but only 2 out of 5 inoculated horses that could be sampled developed a > 4-fold rise in neutralizing antibody titers during the course of the study.

**Table 2:** Titer of viral neutralizing antibody against EHV-5 isolates A and B.

Horse	Day 0, Virus A	Day 49, Virus A	Day 0, Virus B	Day 49, Virus B
C1	64	ND	16	ND
C2	128	ND	64	ND
E1*	≥1,024	ND	256	ND
E2	256	≥1,024	64	128
E3	128	512	32	256
E4**	64	256	32	128
E5	128	256	8	32
E6	32	512	<8	128

The sera were collected before infection on day 0 and on day 49 after infection.

* Horses E1, E2, and E3 were infected with EHV-5 isolate A

** Horses E4, E5, and E6 were infected with EHV-5 isolate B

ND; not done

### Gross Pathology

Gross lesions were restricted to the lungs; lesions were found in 3/6 experimentally inoculated horses. The remaining 3 experimentally inoculated horses, as well as the two control horses, lacked discernable gross abnormalities on postmortem examination. Two horses with gross lesions were infected with virus B and 1 horse was infected with virus A. The gross pathology (Figure 1) was similar among the three horses, and shared similarities to horses naturally affected with spontaneous EMPF. Multifocal nodules of parenchymal fibrosis were present within both the right and left lungs (Figure 1). Lung fibrosis was present within the sub-pleural lung as well as within the deeper parenchyma, and was distributed in both cranial and caudal lung fields. Similar to the spontaneous disease, the nodules were often present as distinct nodules surrounded by grossly normal lung, and varied in size from 1-8cm (Figure 1).

### Histopathology

**Figure 2:**
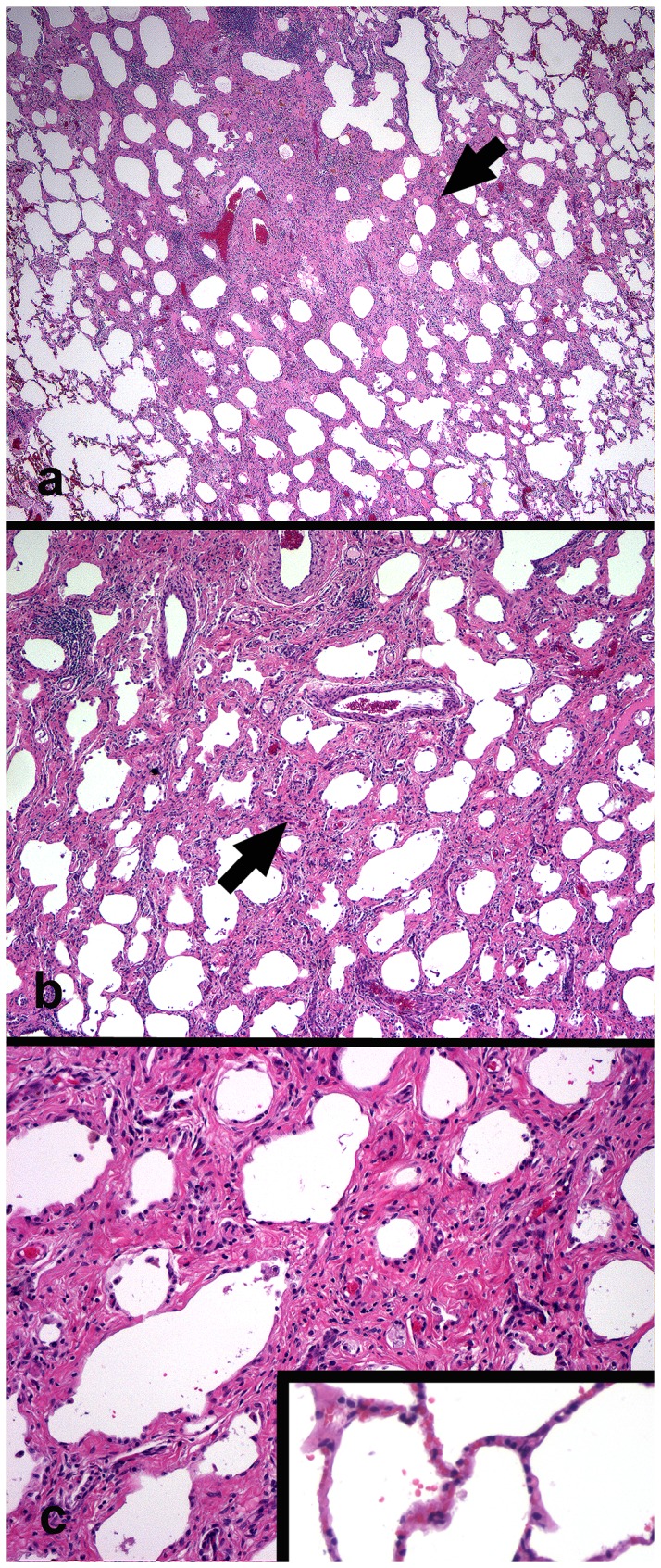
Experimental EHV 5 infection; histopathology (horse E4, C1). Nodules of fibrosis (horse E4 arrows, A, B) were present within the alveolar parenchyma of infected horses. The collagen (pink) disrupted the normal alveolar architecture; occasional foci of lymphocytic inflammation are evident. Higher magnification of the fibrosis with interspersed small blood vessels and mild lymphocytic infiltrates (C). Inset (C) shows normal alveolar architecture of control horse lung (C1). Magnification: A - 2x; B - 10x; C - 20x.

Significant histologic findings were restricted to the pulmonary system, and were found in 5/6 EHV 5 inoculated horses. The severity of the histologic lesions varied with the length of time that had lapsed between infection and euthanasia. The most dramatic findings were in horses that were euthanized between 105 and 108 days post inoculation. Those findings consisted of multiple nodules of fibrosis (Figure 2). The fibrosis was found mostly within the alveolar parenchyma, and consisted of discrete collagenous nodules within the alveolar interstitium, which thickened and disrupted the affected alveoli. Significant regions of fibrosis were also present within the paraseptal alveolar interstitium and interlobular septa. Moderate numbers of small arteriolar vascular profiles were often present within the nodules of lung fibrosis. Inflammation within these nodules was modest, and mostly consisted of small to moderate numbers of interstitial lymphocytes and macrophages, as well as small numbers of hemosiderophages; as in the spontaneous disease, occasional foci of neutrophils and macrophages were present within the remaining airspaces. Viral inclusion bodies were not seen within the lungs of any of the horses. In the horses that were euthanized on day 98 post-inoculation, there was significantly less fibrosis. The changes within these lungs were comprised primarily of small intra-alveolar and interstitial aggregates of lymphocytes and macrophages (not shown).

**Figure 3:**
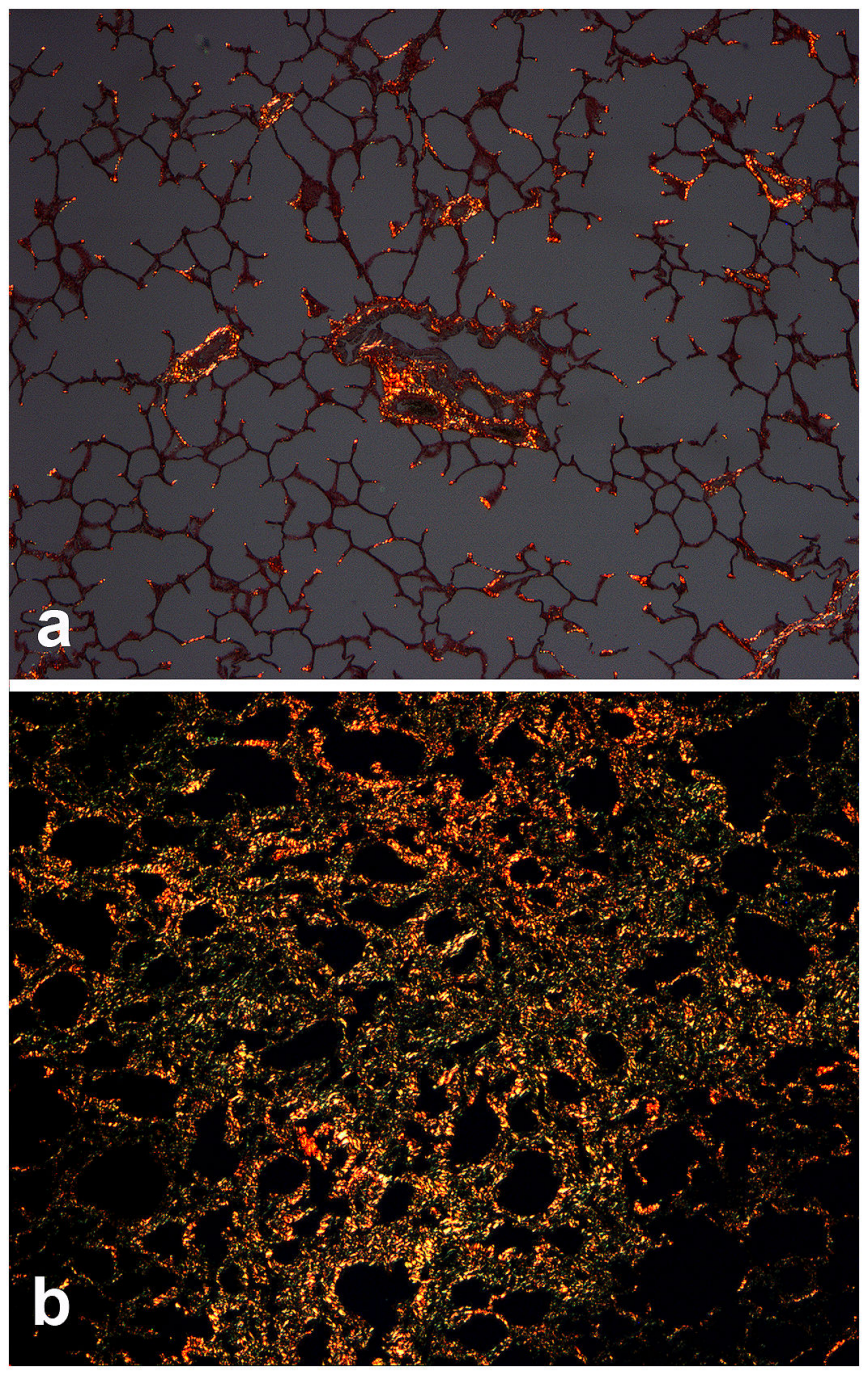
Experimental EHV 5 infection; polarized light microscopy, picrosirius red histochemistry (horse C1, E1). The collagen-specific dye, picrosirius red binds to collagen and fluoresces under polarized light. Within the lung parenchyma, collagen within control horse lung (C1) is restricted to branch points of alveoli and surrounding airways and blood vessels (A). Collagen is significantly increased in the alveolar parenchyma of the lungs of EHV 5 infected horses (horse E1, B). Magnification: A, B - 10x.

To evaluate the distribution and abundance of collagen, serial histologic sections of lung from control and EHV 5 inoculated were histochemically stained with the collagen specific stain pircrosirius red. In the control horses collagen was detected around blood vessels and airways, with small foci of collagen detected at alveolar branch points (Figure 3a). In EHV 5 inoculated horses collagen was detected within the nodules of fibrosis. The collagen was present within the alveolar intersitium and disrupted the alveolar architecture (Figure 3b).

### α- Smooth Muscle Actin Immunohistochemistry for Myofibroblasts

**Figure 4:**
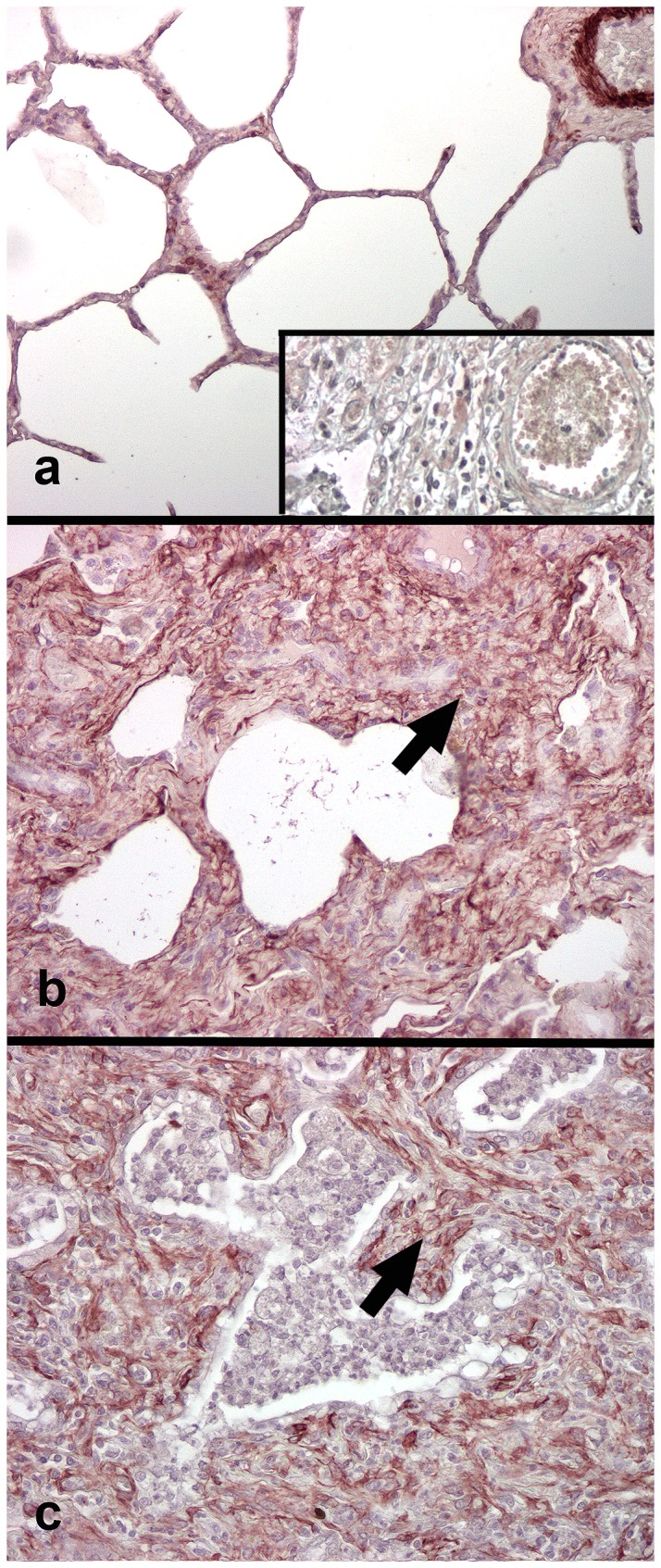
Immunohistochemistry for α-smooth muscle actin for myofibroblasts (MFB) (horses C1, E4). In control horse lung smooth muscle actin is present around blood vessels and within occasional alveolar interstitial MFB (A). Numerous MFB are present within the nodules of alveolar fibrosis in the lungs of EHV 5 infected horses. Similar distribution of MFB is present within the foci of alveolar fibrosis in horses with naturally-acquired EMPF (C). Inset (A) negative control, mouse serum IgG substitution for primary antibody. Magnification: A-C - 20x.

Detection of α-SMA-labeled cells within the lungs of the control horses was restricted to smooth muscle associated with conducting airways, as well as the tunica media of the vasculature (Figure 4a). Myofibroblasts (MFB) were detected within the lungs of all experimentally infected horses. Numerous MFB were present within the alveolar walls of lung regions that were mildly affected. The numbers and density of MFB increased commensurate with the degree of interstitial fibrosis, and in the most severely fibrotic and remodeled lung they were present within the interstitial collagen, as well as immediately beneath epithelial cells lining the airspaces (Figure 4b). The distribution of MFB in the EHV 5 infected horses was very similar to that found within the lungs of spontaneous EMPF (Figure 4c).

### EHV 5 Immunhistochemistry

**Figure 5:**
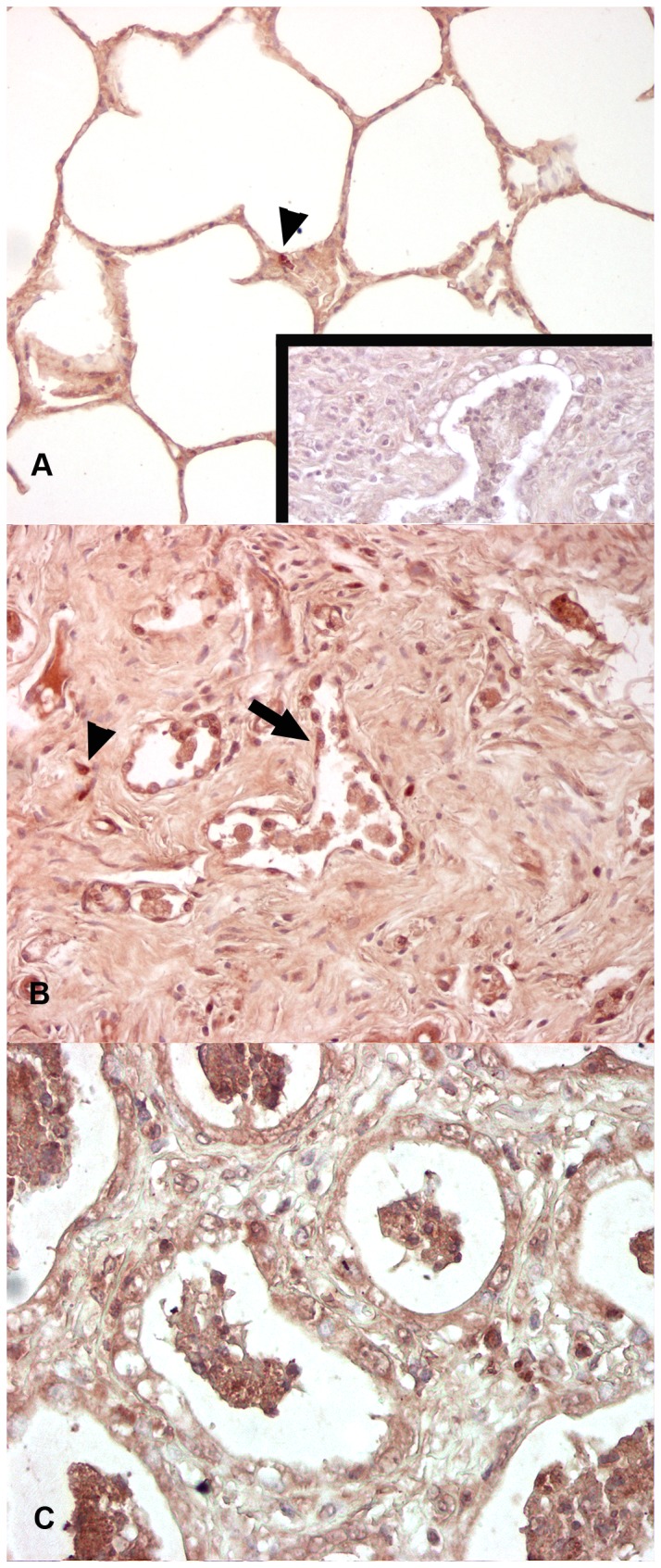
Immunohistochemistry for EHV 5 (horse C2, E4). Control horse (C2) has detectable EHV 5 antigen within scattered alveolar macrophages (arrowhead). EHV 5 infected horses (B) had detectable EHV 5 antigen within the nodules of fibrosis, including the honeycomb epithelial cells (arrow), interstitial fibroblasts, and macrophages. Similar distribution of EHV 5 was found in the lungs of naturally-acquired EMPF (C). Inset (A) negative control, rabbit serum IgG substituted for primary antibody. Magnification: A-C - 20x.

**Figure 6:**
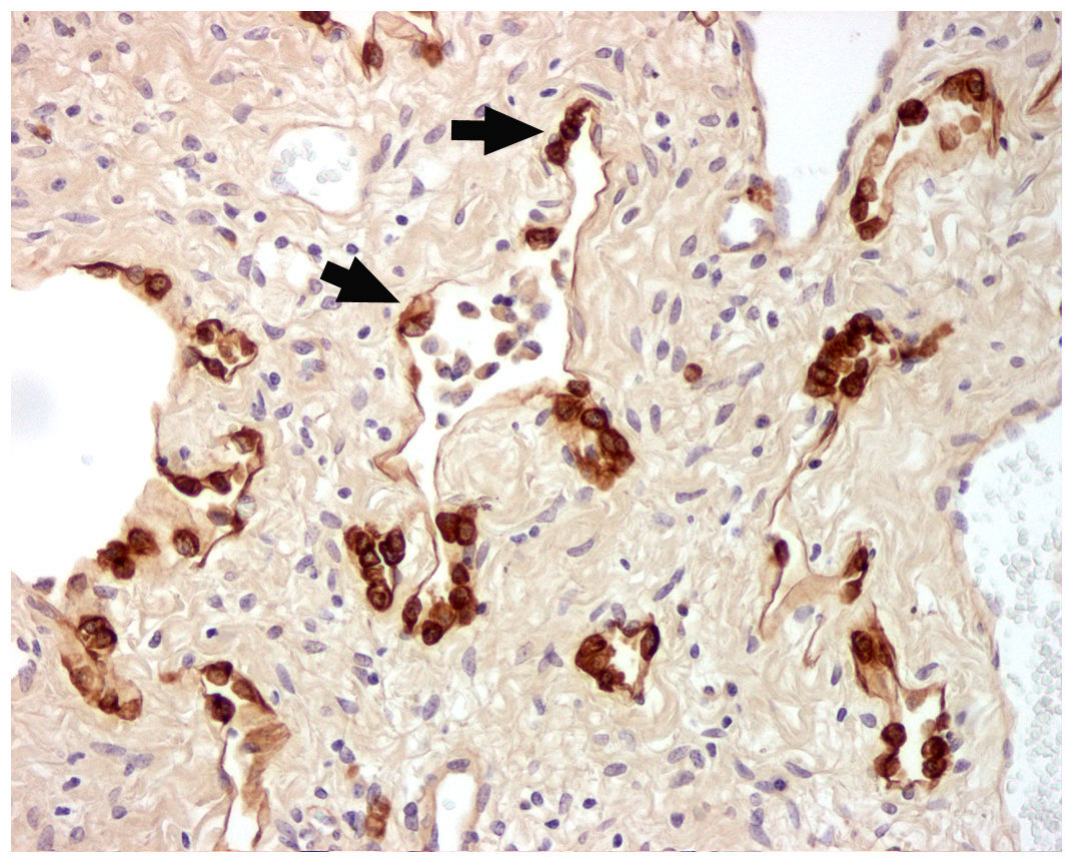
Immunohistochemistry for cytokeratin (horse E4). Cytokeratin expression within the cells of the honeycomb regions of lung (arrows) in the regions of nodular fibrosis in EHV 5 infected horses confirms their epithelial identity – the same cells expressing EHV 5 antigen in Figure 5B (arrow). Magnification 20x.

Rabbit polyclonal serum raised against EHV 5 detected EHV 5 antigen within occasional alveolar macrophages within the lungs of control horses (Figure 5a). There were no significant differences in location of EHV 5 antigen between C1 and C2, in spite of the persistent presence of EHV 5 within blood and tissues of C2 by PCR. The distribution of EHV 5 antigen was similar in spontaneous EMPF lung and the lungs of animals experimentally inoculated with EHV 5. Antigen was detected in both populations within bronchiolar epithelium, honeycomb epithelial cells, macrophages, and interstitial fibroblasts in the regions of fibrosis (Figure 5b/c). The epithelial identity of the antigen-bearing honeycomb epithelial cells was confirmed using immunohistochemistry for epithelial cytokeratin intermediate filaments (Figure 6).

### Lung Collagen Analysis

**Figure 7:**
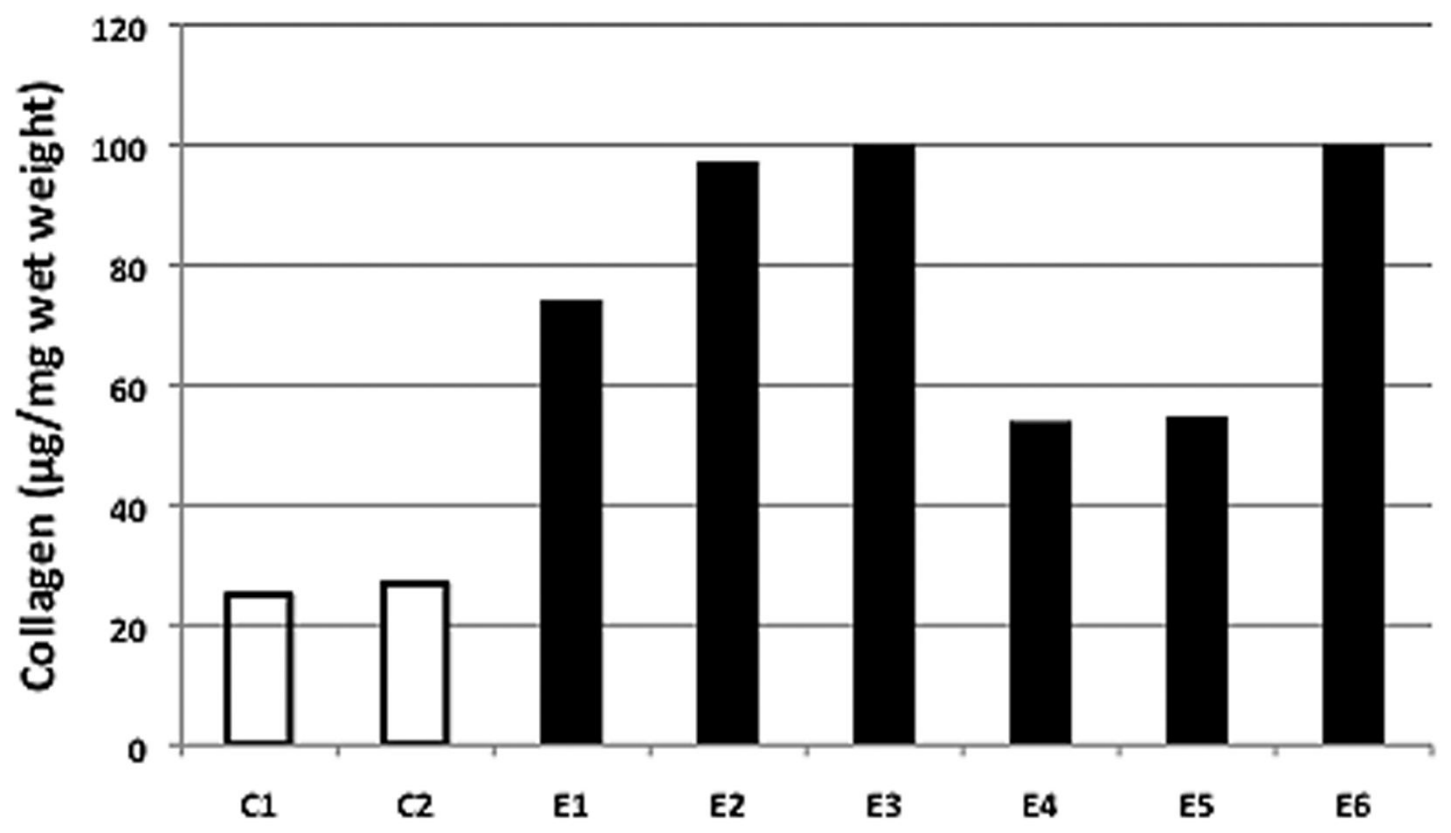
Lung collagen concentration. Collagen concentration (µg/mg wet tissue) was determined for all horses using the Sircol assay (Biocolor Ltd, UK) in control (C1, C2) and EHV 5 inoculated (E1-E6). Collagen concentration was increased in all EHV 5 infected horses. Mean collagen was significantly increased (80 µg/mg) in EHV 5 inoculated horses compared to controls (26 µg/mg) (*p* < 0.5).

**Figure 8:**
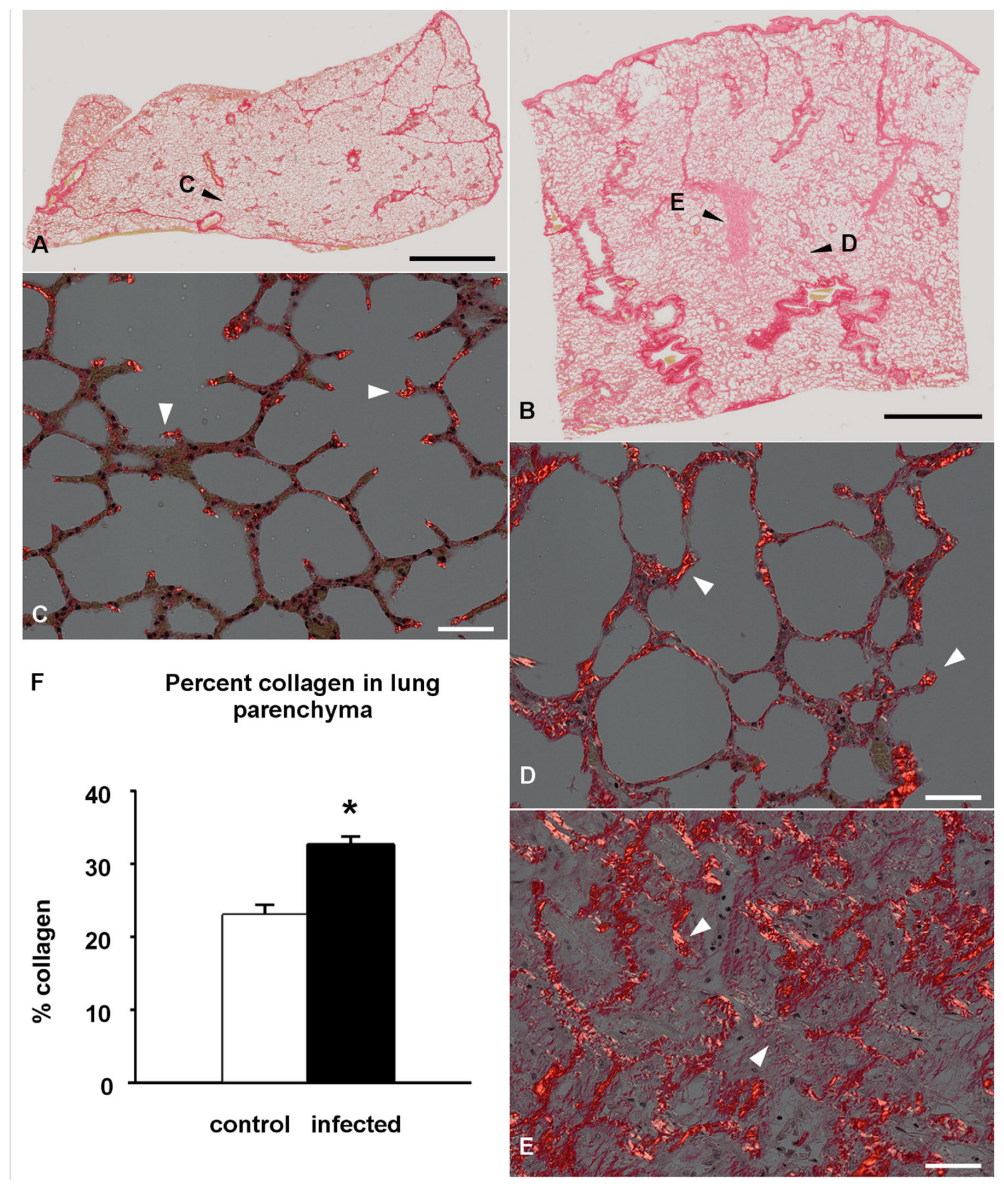
Morphometry for interstitial lung collagen (horse C1, E1 shown). Picrosirius red stained control horse (A) and EHV 5 infected (B); brightfield microscopy. Representative regions analyzed control horse lung (C, shown in ‘a’ above) and EHV 5 infected lung (D, E, shown in ‘b’ above); polarized light microscopy. EHV 5 infected horses had a significant increase in percent collagen within the alveolar parenchyma (F). Magnification: A, B - 2x; C-E - 20x.

Lung collagen was quantified using the Sircol assay (Figure 7) as well as morphometry (Figure 8), Lung collagen concentration averaged 26 µg/mg wet weight in the control horses and 80 µg/mg in the lungs of the EHV 5 inoculated horses (Figure 7). The average lung collagen concentration in the inoculated horses is actually higher than reported, as in two horses (E3 and E6) the determined concentration exceeded the maximum value of the standard curve (100µg/mg). 

In vivo collagen was visualized in histologic sections by staining with the collagen specific dye picrosirius red. Two representative lung sections, representing regions analyzed in a control (Figure 8a) and infected horse (Figure 8b) are shown in Figure 8, as well as a selected area showing the parenchyma at higher magnification and under polarized light (Figure 8C and 8D). Note the focal nodular accumulation of collagen within the lung parenchyma of the infected (Figure 8D) compared to the control horse (Figure 8C). The collagen content of such regions of the lung parenchyma was further quantified by morphometry. As shown in Figure 8E, the alveolar collagen content was significantly increased in the lungs of infected horses (32.6% ± 1.2%) compared to control horses (23% ± 1.4%) (mean ± SEM; p < 0.001).

### Viral isolation and PCR using tissues harvested postmortem

**Figure 9:**
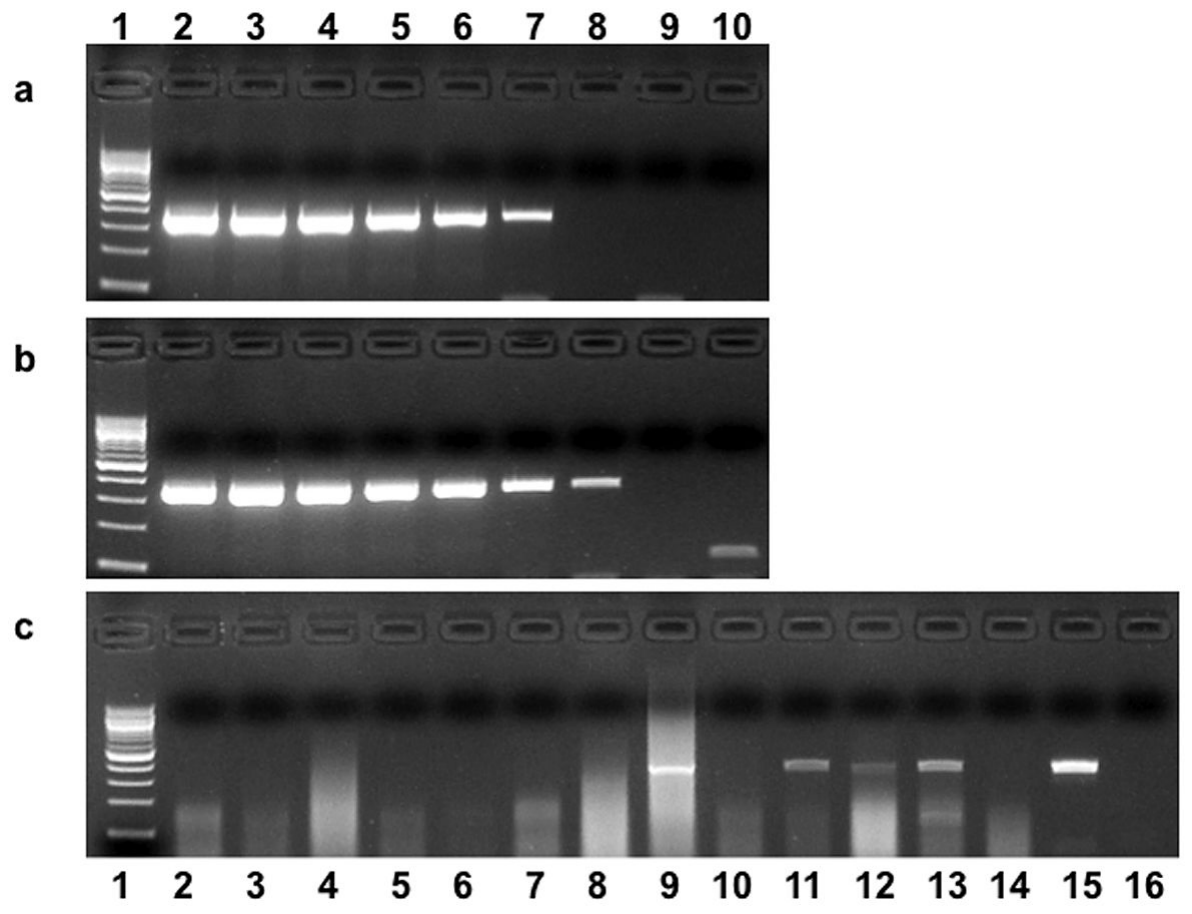
PCR detection of EHV 5 DNA (gH gene) from cell culture and equine tissues (Horse E4). PCR amplicons from serial 10 fold dilutions of DNA extracted from 100 µl of stock virus A (A) or virus B (B) grown in RK-13 cells: lane 1 = 100 base pair DNA ladder; lanes 2 -10 = dilutions of viral DNA from 0 to 10^8^. Example of EHV 5 detection in tissues collected postmortem (C). Lane 1 = 100 base pair ladder; lane 15 = EHV 5 positive control representing approximately 100 PCR detectable units of viral DNA; lane 16 = negative control. Tissue PCR results: lane 2 (nasal mucosa); lane 3 (right caudal lung lobe); lane 4 = mesenteric lymph node; lane 5 = spleen; lane 6 = liver; lane 7 = kidney; lane 8 = tracheobronchial lymph node; lane 9 = left caudal lung lobe; lane 10 = accessory lung lobe; lane 11 = submandibular lymph node; lane 12 = bone marrow; lane 13 = right cranial lung lobe. . Nucleic acid sequence analysis failed to verify that all of the amplicons were from EHV 5 inoculated into the horses (see Table 3).

Virus was not isolated from any tissues collected postmortem from the control or experimentally inoculated horses (Table 3). The limits of detection of EHV 5 DNA by PCR was determined using serial 10-fold dilutions of cell culture isolates of virus A and B (Figure 9a, 9b). The DNA from EHV 5 was not detected in any of the tissues collected postmortem from control horse (C1). Control horse C2 had detectable concentrations of DNA from EHV 5 in the bronchiolar lymph node, submandibular lymph node and kidney on postmortem examination. Nucleic acid sequence analysis indicated the EHV 5 detected postmortem was the same EHV 5 that was detected repeatedly in PBMC from that horse during the study (Table 1). Of the horses that developed gross pathology in the lung (E3, E4, and E5), viral DNA was detected only in lung tissue from E3; however, nucleic acid sequencing indicated that the virus detected was not one of the viruses used for inoculation. DNA from EHV 5 other than the viruses used for inoculation was detected in the bronchiolar lymph node from 2 horses, the submandibular lymph node from 2 horses, the nasal mucosa from 2 horses, the spleen from 1 horse, the mesenteric lymph node from 1 horse and the kidney from 2 horses. Only the bronchiolar lymph node from 1 horse contained DNA from a virus used for inoculation. See Figure 9c for an example PCR gel of postmortem tissue screening for EHV 5 in horse E4.

**Table 3:** Tissues were positive for EHV-5 using PCR assays followed by nucleic acid sequencing.

Tissue type	C1	C2^a^	E1^*^	E2	E3	E4**	E5	E6
Bronchiolar lymph node	−	V_C_	V	−	−	B	−	−
Submandibular lymph node	−	V_C_	V	−	−	−	−	−
Mesenteric lymph node	−	−	−	−	−	−	−	V
Lung	−	−	−	−	V	−	−	−
Nasal mucosa	−	−	V	−	V	−	−	−
Kidney	−	V_C_	−	−	−	−	V	−
Spleen	−	−	−	V	−	−	−	−

The time from infection to post mortem examination were 14 days for C1, 97 days for C2, 98 days for E1 and E2, 105 days for E4 and E6, and 108 days for E3 and E5.

^a^ Horse C2 was naturally infected with an EHV-5 that was designated as V_C_. This horse was in contact with other horses in the study before separate housing was initiated.

* Horses E1, E2, and E3 were experimentally infected with an EHV-5 designated as A

** Horses E4, E5, and E6 were experimentally infected with an EHV-5 designated as B -; negative; V: several different EHV-5 were detected in blood from the horses, those viruses are collectively designated as V

## Discussion

Viruses have been implicated recently in the pathogenesis of idiopathic pulmonary fibrosis[5,27,28]. The suggested role that viral infections might play in IPF include: 1) as the primary initiating event; 2) as co-factors in a ‘multi-hit’ event in the lung to drive the fibrosis; and 3) in precipitating acute exacerbations of disease[5,9,11]. Evidence linking viral infections to pulmonary fibrosis in humans is mostly based on tissue surveillance studies, screening lung samples from IPF-affected individuals. The studies used PCR, with or without immunohistochemistry and in situ hybridization, to identify the presence of viral nucleic acid or proteins in the lung as an indication of viral infection[4,5,7,27–30]. At this time there is no proven direct cause-effect relationship established between viral infection and lung fibrosis in humans or other species. Members of the Herpesviridae family, especially those of the γ-HV subfamily, are most commonly identified within the lungs of IPF patients[4]. The human γ-HV EBV is most commonly associated with IPF, and is considered the virus most likely associated with the development and progression of IPF[6,7]. Humans are the natural host for EBV, yet relative to the high rate of infection with the virus development of pulmonary fibrosis is a comparatively rare event, and the potential for this common γ-HV infection to play a role in the development of pulmonary fibrosis remains unknown. In particular, it is not known if virus infection alone is sufficient to induce lung fibrosis in people. 

 Recently we used virus isolation and PCR assays to detect the equine γ-HV EHV 5 in a cohort of horses first identified with EMPF[14]. Using a systematic nested PCR approach, we identified EHV 5 in lungs from 100% of 24 EMPF-affected horses and in none of the lungs from 23 control horse lungs[14]. This level of association between a virus and a progressive fibrosing lung disease is higher than what is reported in human cases of pulmonary fibrosis, but as in humans it was only an association. Thus we designed the current study to test the potential for EHV 5 to induce pulmonary fibrosis similar to that which develops in EMPF. Similar studies are not possible in people, for obvious reasons, and the murine γ-HV MHV 68 does not naturally infect laboratory mice (*Mus musculus*)[12,13], therefore the horse provides an important opportunity to investigate the potential of γ-HV to induce lung fibrosis in an outbred population of animals from the host species.

We were unable to to use horses for experimental infection without detectable neutralizing antibody (NA) titers against at least one of our isolates of EHV 5. This finding is not surprising given that EHV 5, like EBV in humans, is a ubiquitous subclinical infection and detection of the virus and antibodies to EHV 5 is commonplace in horses[21,31,32]. Realizing that we would be attempting to induce a repeat infection in the face of an existing immune response, we selected horses for the study that had the lowest titers of antibody that reacted with antigen from EHV 5 and antigen from the related equine γ-HV EHV 2. In order to quickly screen horses for antibody, we used an immunoperoxidase staining assay on cell cultures infected with either EHV 5 isolate B or a field isolate of EHV 2. Once the horses were selected, we performed VN tests against both of the EHV 5 isolates that were subsequently used to inoculate the horses. The γ-HV, with their propensity to establish latency, are mostly of low acute pathogenicity, thus they often do not elicit a vigorous neutralizing antibody response[33,34]. There is some thought that VN titers below 1:250 to 1:500 may not protect against reinfection[35]. Most of the horses selected for the current study had relatively low VN titers before experimental inoculation with EHV 5 to suggest they would be susceptible to re-infection. The VN titers increased in all horses after experimental inoculation, indicating infection with EHV 5 had occurred. The evidence from this experiment suggests that the ability of the virus to gain access to the lung may be an important determinant in the pathogenesis of a disease caused by a virus that has heretofore been considered largely non-pathogenic in the natural host. Alternatively, given that control horse C2 consistently had detectable EHV 5 within peripheral blood and tissues but did not develop similar disease, the data suggests that not all strains of EHV 5 are pathogenic, and that there are a few strains of the virus that are capable of inducing lung fibrosis.

Despite repeated attempts, EHV 5 was not isolated from antemortem or postmortem samples from any horse (including C2) and the inoculated strains of EHV 5 seldom were detected using PCR assays. The difficulty to isolate or detect the virus in the inoculated horses is in contrast with our experience in horses with the spontaneous disease[14]. From this, we hypothesize that EHV 5 present within the lungs of horses with spontaneous clinical disease was likely in a lytic phase of infection and in the current study, virus in the lungs of horses with lung fibrosis, but without clinical signs of disease, was present in very low numbers or is in a latent phase of infection. During a latent phase of infection, viral DNA would have been present at very low copy numbers, which prevented detection with the assays used. This finding has important implications when considering detection of γ-HV in the lungs of humans with pulmonary fibrosis, suggesting that the prevalence of detection of γ-HV infections may be underestimated in people depending on the phase of viral infection, i.e. lytic verses latent, and the stage of clinical disease in the patient. It also raises questions regarding tissue levels of virus necessary to participate in lung fibrosis, and whether latent virus or replicating virus is involved in lung fibrosis in the infected animals. The status of γ-HV infection, i.e. active, latent, or reactivation of latent virus, may be important in determining the extent of lung fibrosis that develops during viral infection. Evidence in mice suggests that MHV 68-induced lung fibrosis in INF-γR^-/-^ mice occurs following the lytic phase of infection. Further, preventing latent virus reactivation reduces lung fibrosis[36] , although preexisting latent virus may exacerbate fibrosis following exposure to additional lung injury[11]. 

In the current study the lung pathology was similar between horses experimentally inoculated with EHV 5 and spontaneous EMPF [14], suggesting strongly that the strains of EHV 5 used in the current study were the cause of the lung pathology. The pathology of spontaneous EMPF as well as the nodular fibrosis detected in the EHV 5 inoculated horses differs from usual interstitial pneumonia (UIP), the histopathologic lesion of IPF[37–39]. UIP is typified by the presence of fibroblast foci, temporal heterogeneity (mature fibrosis interposed with ‘younger’ ongoing fibrosis), scant inflammation and the presence of ‘honeycomb’ change[38,40,41]. Fibroblast foci and temporal heterogeneity are not discernable features of the equine disease, indeed the fibrosis in both the spontaneous and experimentally-induced lung fibrosis appears to be of relatively uniform maturity suggesting that lung injury and fibrosis commences during a relatively short time interval (in this experiment, approximately 100 days). The amount of collagen detected within the lungs of EHV 5 inoculated horses was significantly increased compared to control horse lung, and the fibrosis co-localized with numerous myofibroblasts within the alveolar parenchyma. Myofibroblasts are important effector cells in progressive fibrosing lung diseases[42,43], and doubtless are vital to the progression of disease in the EHV 5 infected equine lung.

 How EHV 5 might induce pulmonary fibrosis in the lung of infected horses is not known. Using immunhistochemistry we detected EHV 5 antigen widely within the lungs of infected horses, including in bronchiolar airway epithelial cells, macrophages, and interstitial fibroblasts. This distribution of virus is similar to reports of EBV within the lungs of IPF patients, where virus was detected within bronchiolar and alveolar epithelial cells, including type II pneumocytes, as well as occasional lymphocytes and macrophages[7,44,45]. The antibodies utilized in the human studies detected proteins associated with lytic EBV infection, suggesting that lytic infections may play a role in perpetuating epithelial injury and fibrosis in the lung. In two of these studies the authors report detecting EBV in several of the lung samples by PCR, consistent with the lytic stage of infection when viral nucleic acid is expected to be more abundant[7,45]. In vitro studies provide potential mechanisms toward EBV infection contributing to the development of human IPF. Lytic infection of human type II cells and A549 cells with EBV upregulated transcription of TGF-β[46]. In a follow up study, A549 cells exposed to TGF-β to induce lytic infection experienced induction of non-canonical Wnt signaling pathways and epithelial to mesenchymal transition (EMT)[47], which is an important pathway to myofibroblast differentiation[48,49]. 

IPF in humans and spontaneous EMPF in horses are diseases of adults[14,50,51]. These findings suggest that there may be host factors present within the adult human and equine lung that predispose it to develop fibrosis in response to injury. Xu et al. documented that senescence-accelerated mice develop increased lung fibrosis and TGF-β along with increased peripheral blood circulating fibrocytes following intra-tracheal bleomycin when compared to wild type mice[52]. To address differences in the response of aged vs young lungs to γ-HV infection, Naik et al. infected young (4 mo) and aged (15-18 mo) mice intra-nasally with MHV 68. The aged murine lung developed increased lung collagen, fibrosis, and TGF-β following infection with MHV 68 compared to the young lung, although there were no differences in lung inflammation or virus clearance between the aged and young lung, suggesting inherent differences in the aged lung that promote fibrosis not dependent on inflammation in response to viral infection[53]. A recent study by Torres-Gonzalez et al. showed that the aged murine lung develops increased fibrosis following infection with MHV 68 associated with increased endoplasmic stress response and type II pneumocyte apoptosis, and induction of profibrotic inflammatory pathways compared to young mice[54]. It would be of interest to investigate differences in the response of aged and young equine cells to EHV 5 to see if similar such responses occur between the virus and its natural host.

Because of the difficulty to detect EHV 5 by PCR or re-isolate the virus in inoculated horses we hypothesize that the antigens detected immunohistochemically represent latency-associated proteins expressed by latent virus, and that this latent virus is driving the resultant lung fibrosis. This conclusion, that EHV 5-induced lung fibrosis can occur, and may begin, during viral latency, without additional known stimuli differs with what has been reported in mice[11]. The lung is a major site of viral latency following infection of mice with MHV 68, with latent virus identified in epithelial cells, dendritic cells, and macrophages[55–58]. Many of the latently infected cells, including alveolar macrophages, express increased amounts of a number of pro-fibrotic mediators, including TGF-β[10]. In the clinical stage of naturally occurring EMPF, EHV 5 is replicating and readily detected by PCR assays targeting structural genes (indicating lytic infection) and virus isolation[14]. Analysis of a single horse with clinical EMPF using quantitative PCR determined that the lung contained the greatest viral load, compared to other tissues, and that there was more virus present in fully developed pulmonary lesions than in less affected lung[59]. These data, as well as data from our earlier study[14], suggest that the low levels of virus present in our experimentally infected animals in the current study reflects viral kinetics present during the early stages of lung remodeling, that initiation of lung fibrosis by the virus can occur with very low amounts of latent virus, and that the virus transitions into a lytic productive infection coincident with progression of the disease. 

In summary, we have demonstrated that experimental lung infection in horses with two separate isolates of the γ-HV EHV 5 induces lung fibrosis that is similar to the spontaneous progressive fibrosing lung disease of horses, EMPF. Our data suggest that pulmonary fibrosis in infected horses is initiated by the presence of EHV 5 within the lung. Considering that DNA from strains of EHV 5 other than those used for inoculation were detected in the lung, the strain of EHV 5 that infects the lung may be critical. This *in vivo* study provides evidence that a γ-HV alone - without additional known lung injury – can induce lung fibrosis in an outbred population of animals from its host species. Studies of the interaction between EHV 5 and equine cells may provide new opportunities to investigate the biology of γ-HV -induced lung fibrosis and the role that such viral infections may play in human lung fibrosis.

## Supporting Information

File S1 **PCR primers and reaction conditions.**
(DOCX)
