# Supplementary material for: Experimental Induction of Pulmonary Fibrosis in Horses with the Gammaherpesvirus Equine Herpesvirus 5
Source: PLoS One. 2013 Oct 11;8(10):e77754. doi: 10.1371/journal.pone.0077754 (PMC3795644; doi:10.1371/journal.pone.0077754)
Supplement: File S1 — PCR primers and reaction conditions. (DOCX) [file pone.0077754.s001.docx]

Supporting information S1 - PCR primers and reaction conditions.

All PCR assays were done using GoTaq Green (Promega). The PCR assay targeting the glycoprotein B gene of EHV 2 was done using forward primer 5ʹ-CAGTGTCTGCCAAGTTGATA-3ʹ and reverse primer 5ʹ-ATGGTCTCGATGTCAAACAC-3ʹ with the following conditions: 94°C 240 s (1 cycle); 94°C 30 s, 55°C 30 s, 72°C 30 s (40 cycles); 72°C 300 s (1 cycle). The touchdown PCR assay targeting the glycoprotein H gene of EHV 5 was done using forward primer 5ʹ-TAACCTCCGCGACACGTTTTCA-3ʹ and reverse primer 5ʹ-TAGACATCACCGCAGAAACCACAA-3ʹ with the following conditions: 94°C 240 s (1 cycle); 95°C 30 s, first cycle 65° C 30 s then -1°C per cycle, 72°C 30 s (5 cycles); 95°C 30 s, 60°C 30s, 72°C 30 s (35 cycles); 72°C 300 s (1 cycle). The touchdown PCR assay targeting the glycoprotein B gene of EHV 5 was done using forward primer 5ʹ-TGATATGACGGCCAGATCACAC-3ʹ and reverse primer5ʹ- CCAACCCCACACCATAGTCT-3ʹ with the following conditions: 94°C 240 s (1 cycle); 95°C 30 s, first cycle 65° C 30 s then -1°C per cycle, 72°C 30 s (5 cycles); 95°C 30 s, 60°C 30s, 72°C 30 s (35 cycles); 72°C 300 s (1 cycle). The nested PCR assay targeting the polymerase gene of herpesviruses was done using first round primers 5ʹ- GAYTTYGCNAGYYTNTAYCC -3ʹ, 5ʹ-TCCTGGACAAGCAGCARNYSGCNMTNAA-3ʹ, and 5ʹ-GTCTTGCTCACCAGNTCNCANCCYTT-3ʹ with the following conditions 95°C 900 s (1 cycle); 95°C 30 s, 46°C 60 s, 72°C 60 s (35 cycles); 72°C 300 s (1 cycle). The second round reaction used primers 5ʹ-TGTAACTCGGTGTAYGGNTTYCANGGNGT-3ʹ and 5ʹ- CACAGAGTCCGTRTCNCCRTADAT-3ʹ with the following conditions: 95°C 900 s (1 cycle); 95°C 30 s, 46°C 60 s, 72°C 60 s (40 cycles); 72°C 300 s (1 cycle). The touchdown PCR assay targeting the DNA packaging gene of equine herpesviruses used forward primer 5ʹ-AACTCCTCSGACCAGACCA-3ʹ and reverse primer 5ʹ-SACCACCTTGTGCATGTTG-3ʹ with the following conditions: 95°C 900 s (1 cycle); 95°C 30 s, first cycle 67°C 20 s then -1°C per cycle, 72°C 60 s (10 cycles); 95°C 30 s, 57°C 20 s, 72°C 60 s (40 cycles); 72°C 420 s (1 cycle).
